# Mental health burdens, typhoon and structural health inequities: evidence from a global perspective

**DOI:** 10.1080/16549716.2025.2599623

**Published:** 2026-01-19

**Authors:** Ruoxi Yang, Yu Huang

**Affiliations:** a Department of Biology and Pharmaceutical Engineering, Wuhan Huaxia Institute of Technology, Wuhan, China; b Carbon Neutrality and Climate Change Thrust, Society Hub, The Hong Kong University of Science and Technology, Guangzhou, China

**Keywords:** extreme climate, mental health disorder burden, secondary disaster, typhoon, social health inequality

## Abstract

**Background:**

Against the backdrop of accelerating climate change and more frequent extreme weather events, typhoon disasters have become a major challenge to mental health. Based on the Social Determinants of Health theory and integrating the Cumulative Disadvantage Model with Structural Causal Influence analysis, this study evaluates how typhoon exposure affects the burden of mental health disorders and how these effects vary with social structural differences.

**Objective:**

To investigate the mechanisms linking typhoon exposure to the burden of mental health disorders, and to quantify the moderating roles of macro-level social structural variables.

**Methods:**

By constructing both main effect and year-on-year difference models, combined with structural equation modelling and multinational panel data, this research quantifies the moderating roles of macro-level social variables, including gross national income, Human Development Index, Gini coefficient, government health expenditure, out-of-pocket health spending, educational attainment, and life expectancy.

**Results:**

Typhoons were found to increase prevalence, incidence, and disability-adjusted life years (DALYs) related to mental disorders, with the strongest impact in the 25–34 age group. High income, education, HDI, and public health investment were linked to greater resilience, while low income, high OOP, and high inequality indicated vulnerability. Secondary disaster frequency and the number of people affected acted as mediators, forming a pathway from ‘typhoon’ to ‘social stress’ to ‘mental disorders.’

**Conclusions:**

Typhoon impacts on mental health are shaped by both direct exposure and structural inequalities. Improving socioeconomic conditions, lowering OOP costs, reducing inequality, and increasing public health investment can strengthen psychological resilience and disaster response capacity.

## Paper Context

**Main findings:** This study reveals that typhoon disasters significantly exacerbate the mental health burden across multiple countries, with pronounced heterogeneity by age and socioeconomic status, and that their impacts are strongly moderated by social structure and institutional resources – addressing a key gap in quantifying structural inequality mechanisms in disaster mental epidemiology.**Added knowledge:** For the first time, this research integrates multinational panel data with multidimensional subgroup heterogeneity models and structural equation modelling (SEM) to quantify the moderating and mediating roles of social determinants of health in the typhoon – mental health relationship.**Global health impact for policy and action:** The findings provide empirical evidence for global disaster response policy, underscoring the importance of advancing universal health coverage, optimizing resource allocation, and delivering targeted mental health interventions for vulnerable populations in the context of climate change.

## Background

In recent years, the global burden of mental disorders has continued to rise, becoming a major challenge for public health systems. The Global Burden of Disease Study 2019 highlights that common mental disorders, such as depression, anxiety, and bipolar disorder, consistently rank among the top five causes of disability worldwide, contributing significantly to non-fatal health loss [1]. This upward trend in disability-adjusted life years (DALYs) and quality-of-life losses underscores the increasing importance of mental health in shaping socioeconomic outcomes. The Lancet Commission on Global Mental Health stresses that mental disorders not only impair individual emotional regulation and cognitive function but also affect labour productivity, social roles, and economic performance [2]. Mental health has thus been redefined as an intersection of multiple inequalities, necessitating a governance approach that integrates clinical medicine with social justice and public policy. Despite this, mental health systems globally face significant structural challenges, including inadequate service access, low rates of diagnosis, and uneven resource distribution [3]. In disaster contexts, individuals exposed to sudden traumatic stressors often develop acute psychological responses, such as panic and stress disorders, which can evolve into chronic mental health conditions if not properly addressed. This leads to profound impacts on family stability, social functioning, and public health systems [4]. In regions with underdeveloped mental health infrastructure and pervasive stigma, these disorders are often overlooked, contributing to what is termed a ‘silent epidemic’ [5]

With the intensification of climate change, the frequency and severity of extreme weather events, such as typhoons, have escalated, posing significant risks to public health systems worldwide. Typhoons, in particular, have become more intense, longer-lasting, and broader in impact [6]. According to the IPCC’s Sixth Assessment Report, the vulnerability of public health systems is increasingly evident, especially in resource-constrained developing countries. The Lancet Countdown report further highlights how extreme weather events destroy infrastructure and trigger secondary crises like floods, power outages, and disease outbreaks, all of which compound the mental health burden [7,8].

Many countries face significant ‘institutional fragility’ in their mental health systems during disasters, marked by weak infrastructure, a shortage of trained professionals, and delayed responses to psychological needs. These weaknesses hinder the timely identification and treatment of post-disaster mental health issues, contributing to long-term public health risks [9]. Typhoons create a dual burden of acute shocks and ongoing psychological stress, further complicated by their periodicity, uncertainty in trajectory, and the diversity of secondary hazards. The predictability of typhoons can also induce prolonged anticipatory anxiety, particularly in regions with weak social support systems, exacerbating mental health outcomes [10,11].

Against this backdrop, systematically assessing the pathways through which typhoon disasters affect population mental health carries significant theoretical value and policy relevance. Despite growing attention, three gaps remain: a scarcity of comparative cross-national evidence on typhoon-related mental-health burden [12]; the systematic underestimation of post-disaster psychological impacts due to the latent and under-recognized course of mental disorders [13]; and limited cross-national analysis of structural heterogeneity and mechanisms linking exposure scale and secondary hazards to mental-health outcomes across different social and financing contexts. To address these gaps, we estimate the association between annual typhoon landfalls and country-level mental-health burden (incidence, prevalence, DALYs, and year-over-year changes), assess heterogeneity across demographic groups and national settings, investigate mediating pathways via affected population and secondary hazards, and test for potential non-linear and lagged/cumulative responses.

Building on the objectives outlined above, this study uses a cross-national panel and integrates a time-series design with structural heterogeneity to evaluate the effects of typhoons on mental disorders.

Guided by life-course theory and the social determinants of health, it assesses differentiated impacts across populations and contexts and tests mediating pathways (affected population, secondary hazards) alongside potential non-linear and lagged responses.

The findings provide comparative evidence to support targeted, structurally sensitive post-disaster psychological interventions and policy measures to strengthen community health resilience under intensifying climate risks. This study analyse a panel of 86 countries with recorded typhoon landfalls from 2000 to 2019. The exposure is the annual number of landfalls; outcomes are incidence, prevalence, and disability-adjusted life years (DALYs), plus their year-over-year changes (six measures). Models include individual- and country-level covariates to examine heterogeneity across personal characteristics, national endowments, and health-financing structures. Using a sequential modelling strategy, we test moderation by the number of people affected and by the frequency and diversity of secondary hazards and assess potential non-linear dose–response effects.

The structure of this paper is as follows: the first section outlines the research background; the second introduces the theoretical framework and research contributions; the third discusses the research strategy and methodology; the fourth presents the data and analytical results; and the fifth elaborates on the findings and conclusions, along with directions for future improvement.

## Literature review

Mental health disorders, including depression, anxiety, bipolar disorder, and schizophrenia, are a major global public health issue, characterized by high prevalence, relapse rates, and significant long-term quality of life losses [4]. According to the Social Determinants of Health (SDoH) framework, the onset and progression of these disorders are shaped by structural factors, such as income, education, social status, healthcare access, and gender, which influence individuals’ ability to manage stress and maintain psychological resilience [14]. Vulnerable populations often face barriers to information, limited services, and scarce resources, resulting in structural health inequalities. The ‘cumulative disadvantage’ model suggests that early deprivation, such as low education or childhood poverty, has long-lasting negative effects on mental health [15] ‘Long Arm of Childhood’ [16] and Mezzina et al. [17] argue that social vulnerability not only increases the likelihood of psychological trauma but also systematically constrains recovery pathways and access to services for high-risk populations. From a structural perspective, Wind et al. [18] further demonstrate that widening income inequality, declining community trust, and the erosion of social capital are all associated with increased rates of anxiety and depression. The WHO World Mental Health Report (2022) highlights that these inequities are magnified in LMICs, where mental health systems remain chronically under-resourced and where large treatment gaps persist [19].

Typhoons, as climate-driven recurrent disasters, have unique characteristics – such as unpredictability and multiple cascading secondary crises – that exacerbate mental health issues [10]. Their impacts unfold through three main mechanisms: pre-disaster anxiety caused by prolonged warnings and alerts [20], secondary disasters like power outages and flooding that extend stress exposure and reduce resilience [21], and post-disaster service disruption due to infrastructure collapse and healthcare system paralysis [22]. Research shows that typhoons damage infrastructure, incapacitate healthcare systems, and disrupt community support, leading to a transition from acute to chronic mental health disorders [22]. The health burden extends beyond physical injuries to include social disruptions like family separation, displacement, and income loss, all contributing to higher risks of depression, anxiety, and PTSD [23]. In vulnerable areas, these psychological impacts accumulate over time and often manifest with a delay [21]. Studies from LMICs, particularly in South and Southeast Asia, show that typhoons and floods significantly elevate anxiety and depression symptoms, especially among older adults and socioeconomically disadvantaged groups [24,25]. Digital intervention trials in LMICs further demonstrate that innovative technologies may reduce service gaps and improve resilience [26].

Additionally, post-disaster migration, disordered reconstruction, and a lack of institutional response may amplify health risks. Areas with high proportions of elderly residents, dense populations, or individuals with disabilities are particularly vulnerable to severe psychological trauma [27]. Research also indicates that individuals of lower socioeconomic status are more prone to experiencing post-disaster mental health disorders, such as anxiety and depression, whereas those with higher education and better living conditions tend to exhibit stronger psychological resilience [28]. Displacement and disruption of social networks also weaken individuals’ sense of identity and capacity to adapt, increasing psychological strain [29]. While existing studies have explored the relationship between typhoons and mental health, they often focus on single-country case studies and lack cross-national comparisons of how different social structures mediate post-disaster mental health. Current research mainly focuses on individual-level exposure and incidence rates, with limited attention to how institutional and structural factors shape recovery trajectories [30]. Yet, as the WHO guidance on mental health in emergencies (2024) underscores, disasters disproportionately affect people with pre-existing mental health conditions, and the absence of institutionalized response mechanisms deepens inequities [31]. This gap has become increasingly urgent in light of the growing frequency of complex climate-related disasters.

Departing from the prevailing paradigm that primarily analyses post-disaster psychological outcomes through individual-level variables, this study adopts a structural inequality framework and, for the first time, systematically incorporates multiple social structural variables to construct a heterogeneous impact model linking ‘typhoon exposure – social structure – mental health disorders.’ The study identifies patterns of health risk distribution under multidimensional social heterogeneity and explores the unequal recovery processes of mental health through the lens of institutional fragility. Theoretically, this research integrates the cumulative disadvantage model with structural causal inference modelling [32], offering a multi-level and quantifiable approach to the study of health inequality in disaster contexts. It also provides a scientifically grounded, group-specific, and phase-sensitive foundation for designing post-disaster psychological interventions.

## Methods

**Figure 1.**
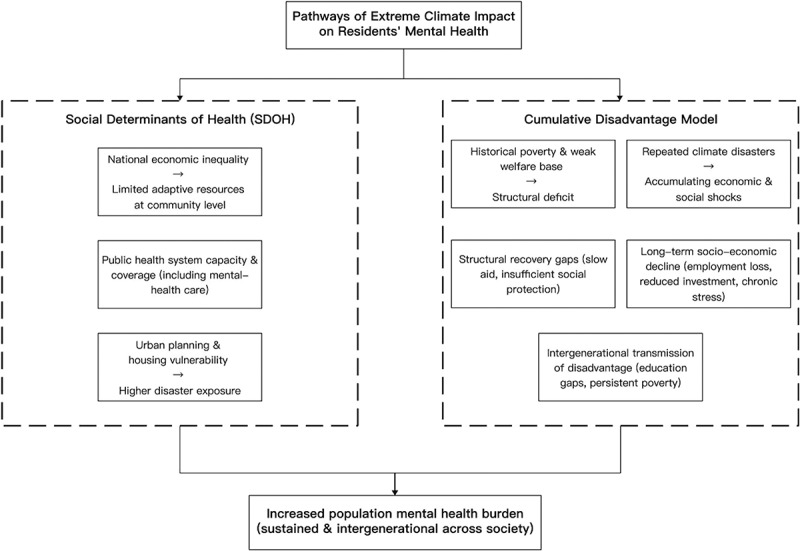
Conceptual framework of typhoon impacts on residents’ mental health.

To investigate the relationship between typhoon disasters and population mental health burden, this study is guided by a conceptual framework that integrates insights from public health, sociology, psychiatry, and disaster management (Figure 1). The framework outlines two complementary pathways through which extreme climate events can shape mental health outcomes. The Social Determinants of Health (SDOH) perspective emphasizes structural conditions – such as economic inequality, health system capacity, and urban planning – that affect the level of community resilience and disaster exposure. In parallel, the Cumulative Disadvantage Model highlights how historical poverty, weak welfare systems, repeated climate shocks, and insufficient recovery mechanisms contribute to long-term socioeconomic decline and intergenerational disadvantage.

Together, these mechanisms explain how typhoon landfalls can generate sustained and intergenerational increases in mental health burden. This conceptual foundation informs the empirical strategy of the study, which employs a two-way fixed effects econometric model to estimate the systematic influence of typhoon events while accounting for socioeconomic covariates, country-specific characteristics, and temporal shocks.

The framework integrates two complementary perspectives: (1) Social Determinants of Health (SDOH), which highlight how inequality, health system capacity, and urban vulnerability constrain adaptive resources and increase disaster exposure; and (2) the Cumulative Disadvantage Model, which emphasizes how repeated climate shocks interact with historical poverty, weak welfare foundations, and structural recovery gaps to create long-term socioeconomic decline and intergenerational transmission of disadvantage. These pathways jointly lead to an increased population mental health burden that is both sustained and intergenerational across society.

### Data selection

This study examines countries that experienced at least one typhoon landfall between 2000 and 2019, with 2019 chosen as the endpoint to avoid confounding effects from COVID-19. Countries with only isolated events, vast land areas, or sparse populations in affected zones were excluded. The final sample includes 85 countries, forming a balanced panel of 1700 country-year observations suitable for baseline and subgroup analyses.

National-level mental health indicators – prevalence, incidence, and disability-adjusted life years (DALYs) – are drawn from the Global Burden of Disease (GBD) study, with year-over-year changes calculated to capture short-term impacts. Disaggregated GBD data by gender and age enable heterogeneity analyses.

Typhoon characteristics (dates, intensity, exposure, secondary impacts) are sourced from EM-DAT. Socioeconomic controls, including GDP per capita (log), unemployment, population density, urbanization, Gini index, income level, HDI, and education, are from the World Bank. Health system indicators – government and out-of-pocket health expenditure shares – are taken from WHO’s Global Health Expenditure Database.

All data management and analysis are conducted in R (v4.3.1), including harmonization of variable definitions, multiple imputation for missing data, and standardization of continuous variables. Two-way fixed-effects models with country-clustered standard errors address serial correlation and heteroscedasticity. Sensitivity checks exclude high-income countries, use alternative controls, and test subgroups by gender and age. Table 1 summarizes all variables.

**Table 1.** Variables’ description.

Variable Type	Variable Name	Unit	Description
Dependent variable	Prevalence	Person	The total number of existing cases of a particular disease or health condition within a population at a specified point in time. This indicator is suitable for assessing the long-term societal burden of chronic mental disorders.
	Incidence	Person	The number of newly diagnosed cases of a specific mental disorder within a defined population over a given period. This measure captures the rate of new occurrences and the spread of mental illness, making it particularly useful for identifying short-term psychological stress induced by disasters.
	DALYs	Person-years	A composite indicator of health loss, calculated as the sum of years of life lost (YLLs) due to premature mortality and years lived with disability (YLDs) due to illness. DALYs reflect the overall impact of a health condition on population-level functioning and productivity and are especially applicable to quantifying post-disaster mental health burden.
	Prevalence_diff		Prevalence in the current year minus prevalence in the previous year.
	Incidence_diff	Person	Incidence in the current year minus incidence in the previous year.
	DALYs_diff	Person-years	DALYs in the current year minus DALYs in the previous year.
Independent variable	Typhoon_Count	Times	Number of typhoon landfalls in the current year.
Subgroup variable	Gender		
	Age		
	HDI		Human Development Index (HDI)
	Gini		Average years of schooling
	Education		
	Income		Income level (per capita classification)
	Gghed		Government health expenditure share (% of total health spending)
	Oop		Out-of-pocket health expenditure share (% of total health spending)
Control variable	Urban		
	Unemployment		
	Intensity		Population density
	Ln(GDP)		GDP per capita (log-transformed)
Mechanism variable	Total_Affect		Total affected population (by typhoon)
	Associated_Freq		Frequency of secondary disasters
	Associated_Type		Number of secondary disaster types

### Model

To quantify the impact of typhoon landfalls on a country’s mental health burden, this study employs a fixed-effects panel regression model. Mental health burden indicators are used as the dependent variable, and the number of typhoon landfalls is used as the independent variable. The model controls for country-specific effects, regional effects, and year effects, using robust standard errors to account for potential heteroscedasticity and serial correlation.

The econometric model is constructed as follows:

(1) $$\mathrm{Dependen}t_{\mathrm{it}}=\beta_{1}\cdot \mathrm{Typhoon}\_\mathrm{Coun}t_{\mathrm{it}}+\beta_{2}\cdot X_{\mathrm{it}}+\alpha_{i}+\gamma_{t}+\varepsilon_{\mathrm{it}}$$

By controlling for these fixed effects, the model allows for a robust estimation of the impact of typhoon landfalls on the national mental health burden while accounting for unobserved heterogeneity and global time trends.

## Result

### Data pattern in typhoon and mental burden indicators

**Figure 2.**
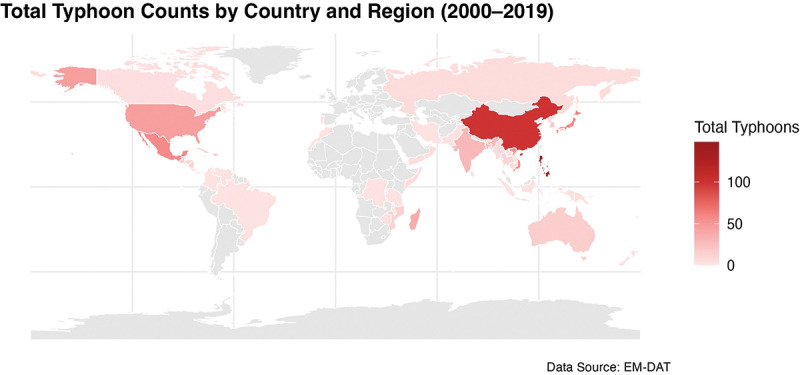
Typhoon counts by country and region (2000–2019).

This study compiles and summarizes data on the cumulative number of typhoon landfalls in countries worldwide between 2000 and 2019 and uses this data to generate a map of typhoon landfalls, as shown in Figure 2. The map reveals significant spatial disparities in typhoon landfalls, with a concentration primarily in East Asia and Southeast Asia.

### Global patterns of mental health burden

**Figure 3.**
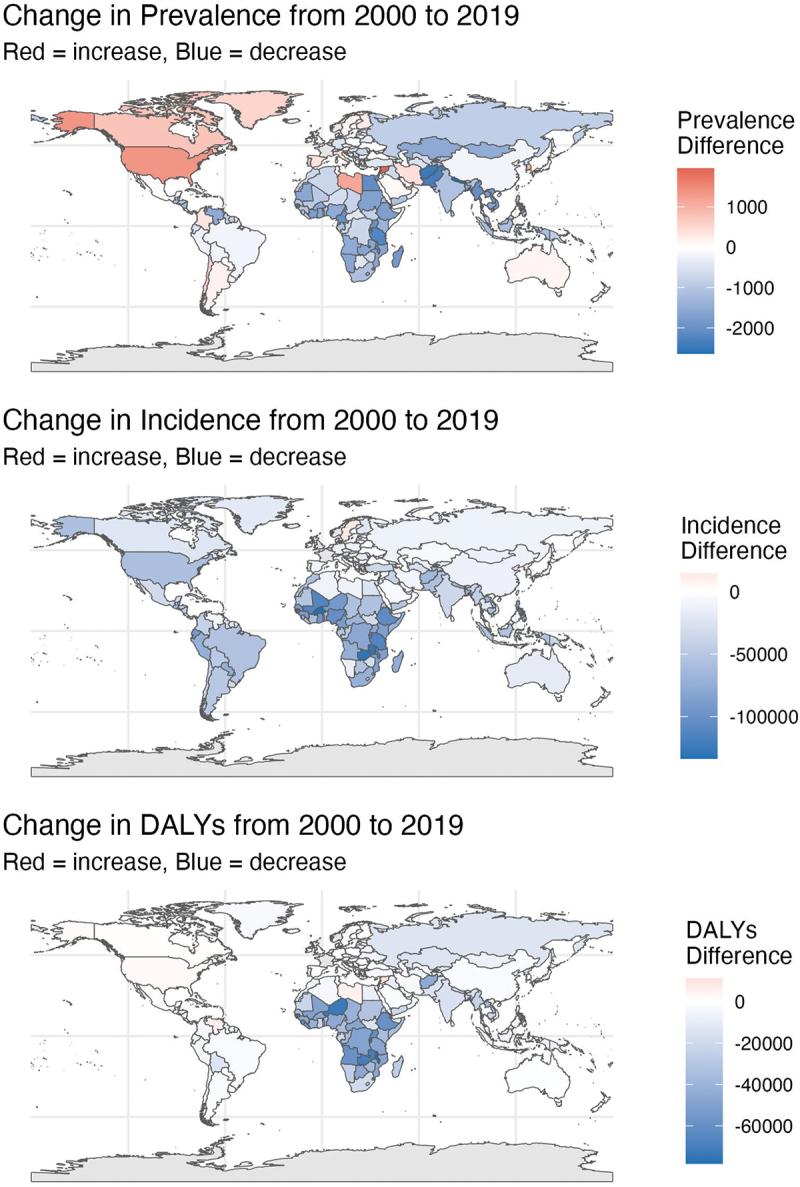
Spatial variation map of mental burden indicators (2000–2019).

This study constructs and analyses spatial variation maps of three mental health indicators – prevalence, incidence, and disability-adjusted life years (DALYs) – globally from 2000 to 2019 (Figure 3). The maps reveal heterogeneous trends: while most countries, particularly low- and lower-middle-income nations, experienced a general decline in mental health burdens, especially in incidence and DALYs, several upper-middle- and high-income countries saw sustained increases across all three indicators, with prevalence rising most consistently. These divergent patterns suggest a nonlinear relationship between national income and mental health burden. Between 2000 and 2019, the global mental health burden generally declined, with notable reductions in low- and lower-middle-income countries. However, several upper-middle- and high-income countries demonstrated increases across all three indicators, with prevalence showing the most consistent rise. Despite the overall global decline, regional variations were observed. Some East Asian countries and high-income Pacific economies recorded limited reductions or even increases in mental health burdens, suggesting that the relationship between national income levels and changes in mental health burden is not linear. Within high-GDP countries, the distribution of mental health burden indicators showed significant disparities across socioeconomic groups. Higher socioeconomic groups generally had better access to diagnosis and treatment, while disadvantaged groups faced persistent barriers to care. This class-based polarization resulted in vulnerable populations bearing disproportionately high levels of unmet mental health needs.

### Main specification: the effect of typhoons on mental burden

Table 2 shows benchmark model results. All independent variable coefficients are statistically positive at the 5% level (Columns (1), (2), (3)), demonstrating that typhoons worsen mental health in afflicted nations. This shows that national mental health burden increases with typhoon landfalls. When considering disability-adjusted life years (DALYs), which measure the population’s mental health over time, typhoons have a major influence. The findings show that typhoons exacerbate mental health issues as external shocks. They worsen psychological discomfort and the mental health crises in impacted countries.

**Table 2.** Benchmark regression.

	(1) Prevalence Number	(2) Incidence Number	(3) DALYs Number	(4) Prevalence Diff	(5) Incidence Diff	(6) DALYs Diff
Typhoon effect	101,164.043**	47,700.438**	19,627.888**	6118.585*	12,423.932*	1181.179*
	(50,808.238)	(18,690.819)	(7782.003)	(3488.764)	(5107.120)	(639.011)
Control variable	Y	Y	Y	Y	Y	Y
Location effect	Y	Y	Y	Y	Y	Y
Time effect	Y	Y	Y	Y	Y	Y
Num. obs.	1700	1700	1700	1615	1615	1615
*R*^2^	0.002	0.004	0.004	0.002	0.004	0.002

*p < 0.1, **p < 0.05, ***p < 0.01.

Standard errors in parentheses.

This study uses the annual difference in mental health burden indicators (diff) as the dependent variable to examine how typhoon landfalls affect national mental health loads in the year of landfall. Table 2 Columns (4), (5), and (6) show the results. Coefficients are also significantly positive at 10%. The cumulative increase in mental health burdens and acute health shocks after typhoons suggest that these impacts may be linked to emotional stress, housing destruction, unemployment, family loss, and social support network failure. Mortality-based public health models are not suitable for mental health research. Mental disorders have low mortality, high morbidity, chronic disease progression, and significant relapse rates, unlike cancer and cardiovascular diseases. Mental diseases have a greater impact on quality of life, functional limits, and disease duration. Instead of death rates, mental health’s ‘burden’ is the number of instances and DALYs lost. Typhoons increase disease prevalence and DALYs, making them a better indicator of post-disaster mental health problems.

Typhoons may raise new cases and lengthen the course of current patients when it comes to mental health. After a disaster, significant environmental changes, loss of life stability, and high psychological stress can cause mental disorders like anxiety, sadness, and PTSD, especially in low-income communities and those who have lost family members. Disasters can disrupt mental health treatments like counselling and medication, slowing patient rehabilitation, causing relapse, or prolonging the disease. The considerable increase in cases and DALYs is due to this ‘increase in incidence + extension of disease course’ dual mechanism. We can conclude that typhoons systematically worsen mental health. In particular, the average increase in DALYs per typhoon, which resulted in nearly 20,000 person-years of loss, shows that post-disaster mental illness is a serious public health issue that can cause functional impairment and long-term health decline.

### Heterogeneity at individual level

**Figure 4.**
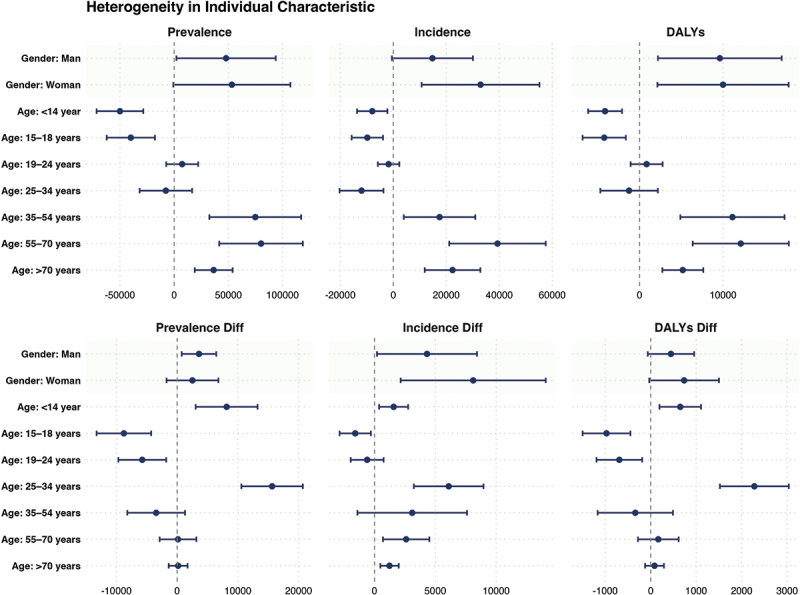
Heterogeneity at the individual level.

The heterogeneity analysis in this study is conducted at both the individual and national levels. Using grouped regression, it systematically examines how factors at the individual level, such as age and gender, and at the national level, such as average years of education (Education Level), Human Development Index (HDI), national income level (Income), life expectancy, Gini coefficient (Gini), government healthcare expenditure share (GGHE-D), and out-of-pocket healthcare expenditure share (OOP), influence the exacerbating effect of typhoons on the national mental health burden. For continuous variables, the sample is divided into two groups: the top 50% as the high group and the bottom 50% as the low group. For variables with missing data in certain countries, these are labelled as ‘Other.’ The heterogeneity effects at the individual level are shown in Figure 4.

This study reveals that typhoons impact mental health through a dual mechanism of ‘increased new cases and extended disease course,’ with effects varying across different age and gender groups.

For children (0–14 years), the overall mental health burden decreases, but the disaster year shows a rise in new cases, suggesting typhoons exacerbate psychological symptoms in the short term. Adolescents (15–18 years) show a significant reduction in all indicators, likely due to delayed diagnosis and underreporting of symptoms, as they are less involved in economic responsibilities. Youth (19–24 years) exhibit a ‘hidden adaptation,’ with decreased mental health burden during the disaster year, possibly due to psychological resilience or lack of post-disaster medical care.

In the 25–34 age group, the mental health burden significantly worsens post-typhoon, driven by family, career, and economic pressures. This group, which is economically active, faces increased stress from job loss and financial instability. The 35–54-year-old middle-aged group shows long-term psychological effects, with a slight decline in new cases but an accumulation of mental health issues over time. For elderly individuals (55–70 years), mental health burdens increase sharply post-disaster, with the highest DALYs, reflecting the compounded effects of chronic illness and slow recovery. The oldest group (70+ years) also shows a significant rise in mental health burden, indicating that cognitive decline, social isolation, and barriers to medical care exacerbate psychological health risks. Gender differences were also observed. Women show a greater increase in new cases and DALYs, likely due to stronger emotional responses, greater caregiving burdens, and a higher likelihood of seeking psychological services. In contrast, men tend to internalize stress, leading to chronic mental health issues that often go unrecognized. In conclusion, these findings highlight the ‘dual mechanism’ through which typhoons impact mental health – namely, the increase in new cases and the extension of disease course – and how this effect varies across different age and gender groups. Women are more prone to acute post-disaster psychological reactions, while men are more likely to develop chronic and underdiagnosed mental health issues. The 25–34-year-old group, due to their central role in social and economic responsibilities, carries the heaviest psychological health burden.

### Heterogeneity at the country level

**Figure 5.**
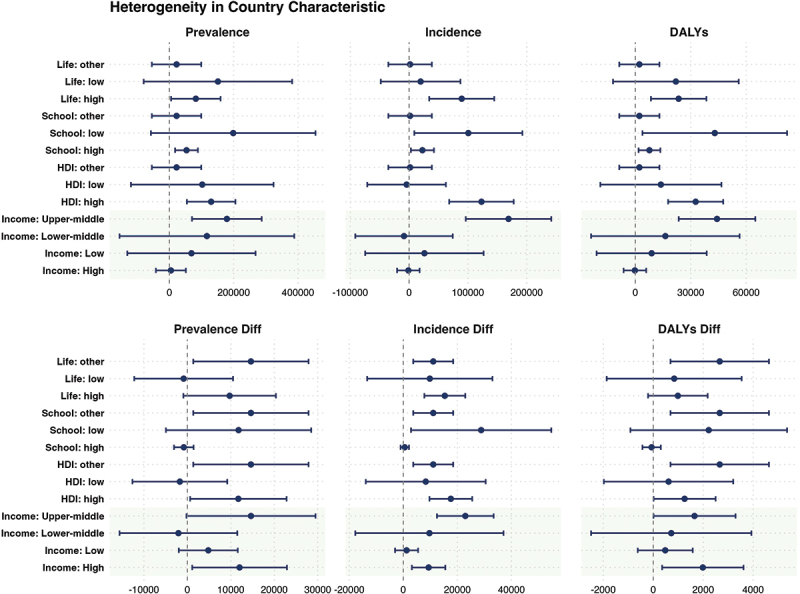
Heterogeneity in the country Level.

The heterogeneity effects at the country level are shown in Figure 5.

This study reveals significant regional and socioeconomic variations in the mental health impact of typhoons. In high-income countries, although the main model shows no significant effects, the diff model highlights a significant rise in mental health burden during the disaster year. This suggests that even with robust healthcare systems, disruptions to daily life and violated expectations can trigger short-term psychological issues. In contrast, low-income countries show no significant typhoon effects, reflecting barriers to healthcare access, symptom expression, and delayed diagnoses.

In middle- and high-income countries, the mental health impact is most pronounced. The main model shows significant increases across all indicators, and the diff model reveals a rise in new cases, reflecting the pressure of urbanization, social transformation, and family responsibilities. For regions with a high Human Development Index (HDI), typhoons significantly raise the mental health burden, even in areas with abundant resources, suggesting that high service accessibility does not equate to stronger psychological resilience. The diff model shows a short-term impact in high HDI regions, indicating that fast-paced lifestyles and social competition amplify post-disaster stress. Conversely, low HDI regions show complex impacts, with no significant changes in the main model, suggesting underdiagnosis and limited mental health recognition. The diff model’s trend direction suggests that mental health issues in these regions often remain unrecognized due to service delays and cultural stigmas.

Countries with higher education levels show a more significant psychological health impact, with the diff model revealing a rise in mental health burden during the disaster year. In contrast, countries with lower education levels experience slower growth in mental health burden, but still face significant risks due to structural disadvantages in healthcare access and recognition.

In high life expectancy countries, typhoons significantly increase mental health issues, with improved recognition and health information systems. The diff model shows a rise in new cases and prevalence, indicating that better recognition correlates with quicker psychological responses. In low life expectancy countries, however, the impact remains hidden, with no significant changes in the diff model, reflecting weak healthcare infrastructure, low mental health service access, and cultural stigma. These countries exhibit a ‘high latency – low recognition’ state, where post-disaster mental health risks are not effectively addressed.

In summary, high HDI and high life expectancy countries tend to exhibit better recognition and intervention, whereas low HDI and low life expectancy countries face significant barriers in identifying and addressing post-disaster mental health needs, which requires stronger service capacity and risk perception.

### Heterogeneity in healthcare expenditure structure

**Figure 6.**
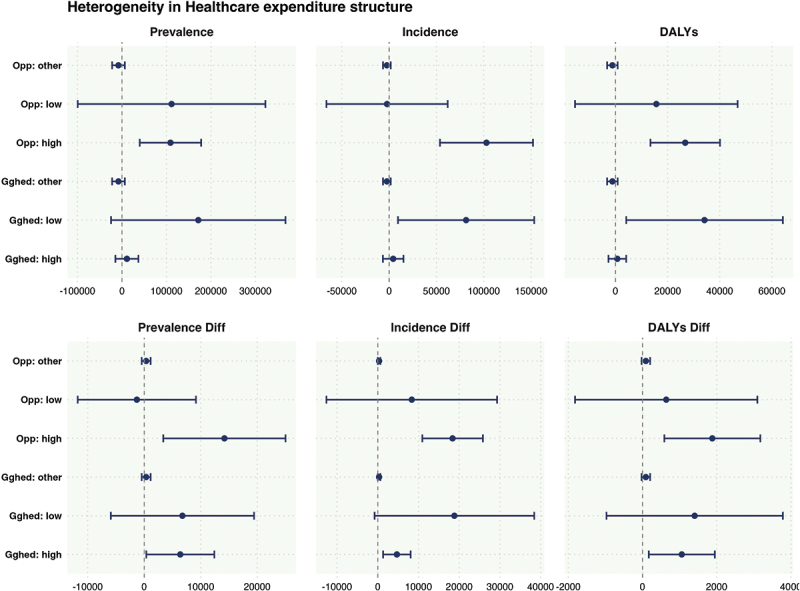
Heterogeneity in healthcare expenditure structure.

This study uses grouped regression to explore the response of key healthcare expenditure structure variables, such as the proportion of government public health expenditure (GGHED) and out-of-pocket healthcare expenditure (OOP), to the mental health burden after typhoon disasters. This reveals the heterogeneity of public and private healthcare systems in disaster response. The analysis is based on the theoretical perspective of ‘Health System Resilience under Disaster,’ aiming to examine how fiscal expenditure structures affect the accessibility, buffering capacity, and equity of post-disaster mental health. The results are shown in Figure 6.

From a government-led healthcare perspective, GGHED is a key indicator of public investment in healthcare, reflecting fiscal capacity for universal health coverage and equitable resource distribution. Empirical results show that regions with lower GGHED exhibit greater psychological vulnerability after a typhoon. The main effect model reveals significant increases in cases, new cases, and DALYs, with the Diff model showing a clear rise in new cases, indicating that the disaster year triggers new mental health disorders. This suggests that in such regions, the post-disaster healthcare system, particularly mental health services, struggles to respond effectively, failing to address both the disaster and its secondary impacts. In contrast, regions with higher GGHED show better resilience, with only the diff model indicating a slight increase in new cases and DALYs, suggesting that high-investment healthcare systems offer some buffering capacity, though psychological stress remains, particularly among vulnerable populations. In countries with high out-of-pocket (OOP) costs, the healthcare system operates differently. In these regions, the mental health burden significantly increases after a typhoon, as high OOP costs create economic barriers to timely treatment and psychological support, exacerbating mental health issues. This may be linked to the economic decline post-disaster and disruptions to medical services and social support systems. On the other hand, regions with lower OOP exhibit a stronger buffering effect, with minimal changes in mental health outcomes post-disaster, except for a slight increase in DALYs. This indicates that lower OOP costs enhance the accessibility of mental health services, reducing the psychological impact and slowing the progression of mental disorders. In summary, GGHED and OOP represent two distinct healthcare system models, reflecting how public and private systems mediate the psychological burden of disasters. High GGHED reflects a robust ‘safety net,’ while low OOP enhances healthcare accessibility. Both factors contribute to the structural resilience and equity of post-disaster mental health services. This supports the applicability of the ‘social determinants of health’ theory in disaster contexts and suggests that policies should focus on optimizing fiscal structures and reducing personal healthcare costs to strengthen mental health interventions during extreme weather events.

### Mechanism analysis

This study uses panel data to estimate the direct effect of the independent variable on the dependent variable, examine how a mediating variable affects this relationship, and test how this mediation effect varies across time periods or groups using a diff model. The analysis examines mechanism variables including total affected population, secondary disaster frequency, and secondary disaster types. The results are presented below.

The results, with the total number of affected individuals as a mediating variable, are presented in Table 3. The analysis demonstrates that the total affected population significantly mediates the relationship between typhoons and mental health burdens. This suggests that the scale of disaster exposure heightens the risk of mental health disorders by intensifying trauma symptoms at both individual and societal levels. The diff model further reveals that an increase in the affected population during the disaster year exacerbates the mental health burden, resulting in cumulative disease losses.

**Table 3.** Mechanism test: Mediator = Total_Affect.

	Mechanism Test: Mediator = Total_Affected			
Outcome	Step 1: Typhoon → Y	Step 2: Typhoon → Mech	Step 3: Typhoon → Y (w/Mech)	Step 3: Mech → Y
Prevalence	101,164.043** (50,808.238)	390,494.629*** (53,440.392)	89,499.767* (51,632.315)	0.030 (0.024)
Incidence	47,700.438** (18,690.819)	390,494.629*** (53,440.392)	62,783.064*** (18,887.119)	−0.039*** (0.009)
DALYs	19,627.888** (7782.003)	390,494.629*** (53,440.392)	19,466.924** (7912.096)	0.000 (0.004)
Prevalence diff	6118.585* (3488.764)	390,494.629*** (53,440.392)	9123.240*** (3520.894)	−0.008*** (0.002)
Incidence dDiff	12,423.932** (5107.120)	390,494.629*** (53,440.392)	14,515.792*** (5183.807)	−0.005** (0.002)
DALYs diff	1181.179* (639.011)	390,494.629*** (53,440.392)	1321.046** (649.378)	−0.000 (0.000)

*p < 0.1, **p < 0.05, ***p < 0.01.

Standard errors in parentheses.

Secondary disasters – such as floods, power outages, infrastructure collapse, and infectious disease outbreaks – act as persistent sources of psychological stress due to their ongoing nature and delayed response. Table 4 demonstrates that the frequency of secondary disasters significantly affects the prevalence, incidence, and DALYs of mental health disorders. These disasters indirectly increase the mental health burden, with typhoons contributing to a higher frequency of secondary crises. The direct effect also remains significant, indicating a dual pathway of ‘direct shock plus indirect amplification.’ The difference-in-differences (diff) model further confirms that, in years characterized by a high frequency of secondary disasters, the mental health burden rises significantly, emphasizing how secondary crises exacerbate the cumulative mental health impact on vulnerable populations.

**Table 4.** Mechanism test: Mediator = Associated_freq.

	Mechanism Test: Mediator = Associated_freq			
Outcome	Step 1: Typhoon → Y	Step 2: Typhoon → Mech	Step 3: Typhoon → Y (w/Mech)	Step 3: Mech → Y
Prevalence	101,164.043** (50,808.238)	0.924*** (0.022)	28,531.843 (72,717.393)	78,575.017 (56,294.791)
Incidence	47,700.438** (18,690.819)	0.924*** (0.022)	54,108.141** (26,765.758)	−6931.985 (20,720.941)
DALYs	19,627.888** (7782.003)	0.924*** (0.022)	10,340.558 (11,139.738)	10,047.225 (8623.924)
Prevalence diff	6118.585* (3488.764)	0.924*** (0.022)	11,763.455** (5035.361)	−6123.491 (3940.433)
Incidence diff	12,423.932** (5107.120)	0.924*** (0.022)	21,703.573*** (7369.626)	−10066.449* (5767.117)
DALYs diff	1181.179* (639.011)	0.924*** (0.022)	1707.049* (922.829)	−570.458 (722.162)

*p < 0.1, **p < 0.05, ***p < 0.01.

Standard errors in parentheses.

The ‘types of secondary disasters’ here specifically refer to various secondary crisis events directly triggered by the typhoon, including floods, landslides, infrastructure collapse, traffic disruptions, power outages, and infectious disease outbreaks. Due to their diversity, sudden occurrence, and varying recovery periods, these secondary disasters often constitute delayed psychological stress sources, which can amplify mental health risks at the population level through the disaster-chain mechanism.

The model estimation results (Table 5) show that, compared to the ‘frequency of secondary disasters’ variable, the ‘type’ variable as a mediating path is overall not significant, demonstrating a weakened mediating effect. This suggests that the type of disaster itself has a limited role in the formation of individual mental health burden, and that the direct exposure to the disaster and perception of stress are more decisive. The results from the diff model also show a consistent trend, further confirming that different types of secondary disasters do not significantly mediate the variation in mental health burden between disaster and non-disaster years.

**Table 5.** Mechanism test: Mediator = Associated_type.

	Mechanism Test: Mediator = Associated_type			
Outcome	Step 1: Typhoon → Y	Step 2: Typhoon → Mech	Step 3: Typhoon → Y (w/Mech)	Step 3: Mech → Y
Prevalence	101,164.043** (50,808.238)	0.421*** (0.014)	1170.499 (62,651.605)	237,686.195*** (87,463.786)
Incidence	47,700.438** (18,690.819)	0.421*** (0.014)	41,382.998* (23,098.785)	15,016.652 (32,246.695)
DALYs	19,627.888** (7782.003)	0.421*** (0.014)	5902.713 (9600.306)	32,624.951** (13,402.356)
Prevalence Diff	6118.585* (3488.764)	0.421*** (0.014)	8253.174* (4308.131)	−5109.288 (6048.788)
Incidence Diff	12,423.932** (5107.120)	0.421*** (0.014)	17,069.534*** (6304.790)	−11119.570 (8852.177)
DALYs Diff	1181.179* (639.011)	0.421*** (0.014)	1343.055* (789.240)	−387.463 (1108.125)

*p < 0.1, **p < 0.05, ***p < 0.01.

Standard errors in parentheses.

## Discussion

Typhoon catastrophes increase mental health burdens in numerous countries, as measured by prevalence, incidence, and disability-adjusted life years. Typhoon exposure and mental health follow a three-stage ‘disaster shock – structural modulation – health outcomes’ pathway, in which social structures and institutional resources moderate the link.

### Heterogeneity across countries and regions

Results revealed substantial heterogeneity. In many low- and lower-middle-income countries, apparent declines in incidence and DALYs likely reflect limited detection and reporting capacity, forming ‘statistical silence zones.’ Conversely, upper-middle- and high-income countries showed sustained increases in prevalence, consistent with a ‘high detection–high reporting’ effect arising from improved diagnostic capacity and awareness. In addition, several East Asian and Pacific high-income economies exhibited limited or even negative reductions, indicating that changes in burden are not linearly associated with macroeconomic level.

### Within-country inequalities

Even in high-GDP settings, the benefits of stronger systems were unevenly distributed. Higher socioeconomic groups had greater access to diagnosis and treatment, while disadvantaged populations faced persistent diagnostic gaps, creating ‘diagnostic silence zones.’ This pattern of class polarisation underscores that macro-level improvements in resources do not automatically translate into equitable outcomes.

### Structural conditions and protective factors

Institutional capacity – including fiscal resources, social protection, and healthcare governance – was closely associated with the scale of impacts. Importantly, countries with universal health coverage (UHC) experienced less severe post-disaster increases, suggesting a potential protective role. This finding aligns with principles of global health equity and emphasizes the importance of structural readiness in mitigating long-term harm.

### Comparison with existing literature

These findings are consistent with prior evidence linking extreme weather events to psychiatric morbidity [33], but this study advances the field in two ways. First, by employing DALYs, it captures population-wide, long-term impacts beyond short-term PTSD or depression outcomes. Second, it highlights how structural and institutional differences explain both cross-country variation and within-country inequalities, extending the scope of disaster epidemiology into the domain of global health inequality.

### Policy implications

The findings of this study highlight the need for context-specific policy responses to mitigate the mental health consequences of typhoon disasters. Strengthening disaster preparedness by embedding mental health indicators into surveillance systems and ensuring the rapid deployment of psychological first aid (PFA) within 72 h has been emphasized as a crucial component of climate-disaster governance [34,35]. For example, in the aftermath of Typhoon Haiyan (Yolanda, 2013), the Philippines adopted an integrated MHPSS approach. Core components were delivery of Psychological First Aid within 72 h via mobile teams; mhGAP-informed task-sharing with short-course training and supervision of frontline and primary-care providers for screening and basic psychosocial care; and tiered paper- and digital-based referral pathways linking barangay health posts with municipal and provincial/tertiary facilities to maintain continuity of care. Subsequent institutionalization through the Mental Health Act (2018) strengthened community-based services, enabled telepsychiatry for follow-up, and introduced disaggregated (sex/age/region) monitoring to identify underserved populations. Collectively, these measures illustrate how early PFA, enhanced frontline capacity, structured referral systems, and equity-focused surveillance can reduce post-typhoon mental-health burden and inform resource allocation.

In middle- and high-income countries, institutionalizing post-disaster psychological care within national insurance schemes and establishing digital referral and follow-up platforms can enhance continuity of care, in line with evidence supporting the scale-up of multi-level interventions in technologically equipped systems [36]. In low-income and highly unequal contexts, priority should be given to expanding access to basic services through frontline training, mobile clinics, and telepsychiatry, while international collaborations can provide standardized intervention packages including essential medicines and psychosocial tools [37,38]. Finally, addressing inequities requires transparent and disaggregated monitoring of service access to identify ‘silence zones,’ as most national climate adaptation policies still neglect mental health, underscoring the urgency of integrating equity-oriented approaches into adaptation agendas [39,40].

### Limitations and future research

This study has several limitations. First, although fixed-effects models reduce confounding, unobserved factors, such as changing stigma or health-seeking behaviours may remain. Second, reliance on national data may underrepresent undiagnosed or untreated cases. Third, cross-country differences in exposure measures limit comparability. Future research should combine individual-level longitudinal data with natural experiments (e.g. UHC expansion), employ high-resolution disaster exposure metrics, and evaluate the cost-effectiveness of interventions, such as PFA and tele-mental health in disaster-prone regions.

## Conclusion

This study elucidates the complex, socially moderated relationship between typhoon disasters and mental health disorders. By employing multi-dimensional heterogeneity models and a three-step mechanism analysis framework grounded in the SDH theory, it confirms that typhoons substantially exacerbate mental health burdens, while the scale and nature of these impacts are significantly shaped by social structures and institutional capacity.

The findings underscore that disaster mental health governance must be **context-specific and equity-oriented**. In particular, universal health coverage (UHC) emerges as a key structural safeguard, associated with more timely and equitable responses.

In the aera of climate change, when extreme weather events are projected to intensify, building resilience into health systems and ensuring fair access to care will be essential to protect vulnerable populations and uphold the right to health globally.

## Data Availability

The data that support the findings of this study are openly available as secondary datasets from the following sources: **Global Burden of Disease (GBD)**, Institute for Health Metrics and Evaluation (IHME), at https://vizhub.healthdata.org/gbd-results/ **Emergency Events Database (EM-DAT)**, Centre for Research on the Epidemiology of Disasters (CRED), at https://public.emdat.be/ **World Bank Open Data**, at https://data.worldbank.org/ **World Health Organization (WHO) Global Health Expenditure Database**, at https://apps.who.int/nha/database All datasets are publicly available, free of charge, and contain aggregated, de-identified data. No individual-level information was used in this study. All data used in this study are publicly available. Source datasets include the Global Burden of Disease 2019 (GBD), the EM-DAT International Disaster Database, the World Health Organization (WHO) Global Health Observatory, and the World Bank World Development Indicators. The constructed country-year panel and full replication package – covering variable-construction code and data-cleaning/analysis scripts – are deposited in OpenICPSR under DOI 10.3886/E237163V1 (Version V1). Detailed instructions to reproduce the results are provided in the repository README. No proprietary or individual-level identifiable data were used.
